# Preparation and Characterization of Composites Materials with Rubber Matrix and with Polyvinyl Chloride Addition (PVC)

**DOI:** 10.3390/polym12091978

**Published:** 2020-08-31

**Authors:** Dan Dobrotă, Valentin Petrescu, Cristinel Sabin Dimulescu, Mihaela Oleksik

**Affiliations:** 1 Faculty of Engineering, Lucian Blaga University of Sibiu, 550024 Sibiu, Romania; valentin.petrescu@ulbsibiu.ro (V.P.); mihaela.oleksik@ulbsibiu.ro (M.O.); 2 S.C. Dedeman S.R.L, 600093 Targu Jiu, Romania; cristi.dimulescu@yahoo.com

**Keywords:** rubber, polyvinyl chloride, accelerated aging, finite element method, materials structure analysis

## Abstract

An important problem that arises at present refers to the increase in performances in the exploitation of the conveyor belts. Additionally, it is pursued to use some materials, which can be obtained by recycling rubber and PVC waste, in their structure. Thus, the research aimed at creating conveyor belts using materials obtained from the recycling of rubber and PVC waste. Under these conditions, conveyor belts were made that had in their structure two types of rubber and PVC, which was obtained by adding in certain proportions of reclaimed rubber and powder obtained from grinding rubber waste. In order to study the effect of adding PVC on properties, four types of conveyor belts were made, with the structure of rubber, PVC and textile reinforcement. These have been subjected to certain mechanical tests, also being analyzed from the point of view of the behavior of the accelerated aging. The results obtained showed that the addition of PVC lead to a decrease in tensile stress for the strips made, but also an increase in the tensile strain. Additionally, the elasticity tests performed before and after the accelerated aging showed that the presence of PVC in the structure of the conveyor belts determined a substantial reduction of the aging process of the rubber in the conveyor belts. Under these conditions, it has been established that the use of PVC in the structure of rubber matrix conveyor belts is beneficial if conveyor belts are to be produced that are less subject to mechanical stress, but that work in conditions that can cause accelerated aging of materials. An analysis with the finite element method (FEM) of the test samples was also performed.

## 1. Introduction

The recycling of rubber waste represents a problem that must have an optimal solution from a technical and economic point of view. Thus, it is necessary to find another way to reduce the waste stock of rubber products and PVC, by making a new material. By recycling these types of materials and adopting appropriate technologies, composite materials with superior characteristics can be obtained. For their development with various applications, 3D printing technology can be used, in these conditions, the modified polymer having a series of advantages associated with rapid prototyping [1]. Thus, this technology can be a solution to obtain high-performance composite materials with rubber matrix and PVC additives. Additionally, the use of polyhedral oligomeric silsesquioxanes (POSS), polyethylene glycol 20,000 (PEG) and halloysite nanotubes (HNT), to the manufacture of polymer composites, allows the combination of polymeric structures with a particular and exclusive chemical composition [2,3]. In present, composite materials with a recycled PVC matrix mixed with plasticized PVC and an armature with grinding rubber from waste have been made, and the results obtained showed that such a composite material has interesting acoustic properties at frequencies higher than 2500 Hz. Additionally, the use of ground rubber waste has led to an improvement in the rigidity of such composite materials with an increase in Young’s modulus, but also to a slow decrease in tensile strength, elongation at break and hardness [4,5,6]. 

A possible variant of making rubber and plastic composite materials refers to the use of various concentrations of epoxidized natural rubber (ENR) solution, respectively, dimethylformamide (DMF), which were added directly in a solution of PVC. The results obtained from the differential thermal analysis (DTA) indicated that the addition of ENR led to glass transition temperature (T_g_). It was also found that the mixture of PVC with ENR is thermodynamically incompatible [7]. In our opinion, this technology of making materials can have a negative impact on the environment, explained by the fact that the excessive use of natural rubber in the structure of composite materials, can cause improper exploitation of rubber tree plantations and thus a degradation of the environment. Under these conditions, to reduce the impact on the environment, natural rubber can be replaced with pectin/cellulose nanocrystals/glycerol nanocomposites [8]. 

Currently, investigations have been made into the use of functionalized acrylonitrile-butadiene rubber (NBR-g-MAH) as an impact modifier and a compatibilizing agent in the recycled mixture of PVC and polymethylmethacrylate (PMMA). Thus, it was found that the performance of NBR as an impact modifier was improved by the addition of maleic anhydride. Additionally, changing the concentration of the NBR-g-MAH/NBR mixture has different effects on the mechanical, thermal and morphological properties of the recycled mixture. Thus, the recycled mixture that has 9% by weight NBR-g-MAH/NBR mixture, has the best impact resistance and elongation at break. Following the DTA and microscopic analysis, partial miscibility was observed, as well as the compatibility of the polymeric constituents in the mixture [9,10,11]. 

In order to increase the performance in the use of rubber and PVC composite materials, the straw fibers (SF) were used. Thus, composite materials of the PVC/SF type coated with liquid nitrile-butadiene rubber (PVC/LNBR-SF) were created. The analysis of the mechanical properties showed that PVC/LNBR-SF type composite material presented a better tensile strength, elongation at break and higher impact resistance than PVC/SF composites, due to the compatibilizing and curing effect of LNBR. At the same time, an improvement of the adhesion to the interface between the SF matrix and PVC with the addition of LNBR was obtained. Under these conditions, research has shown that PVC/LNBR-SF composite materials are a solution for replacing wood in practical applications [12]. Studies on the effect of two types of nanoparticles on the morphology and dynamic-mechanical properties of PVC and NBR mixtures, respectively, were performed. The results of the studies revealed that the tensile strength and modulus of PVC/NBR nanocomposites reinforced with 1 phr of single-wall nanotubes (SWNT), are very close to the case of using a quantity of 5 phr of nanoclay. In the case of PVC/NBR/nanoclay, the fracture surface of the samples was much rougher, while the fracture surface of the original PVC/BNR was very smooth. In the case of PVC/NBR/SWNT nanocomposites, it was found that the introduction of carbon nanotubes in the polymer matrix considerably improved the dispersion and distribution of NBR as a minor phase in PVC [13].

The effects of dynamic vulcanization on the properties of the composite of (PVC)/epoxidized natural rubber (ENR)/(powder of kenaf core) were studied. Thus, it was found that the tensile strength, elongation and Young’s modulus of the composites showed an increase for the samples obtained by dynamic vulcanization. Furthermore, the swelling index indicated that the composite with dynamic vulcanization shows a lower absorption of toluene compared to the composites without dynamic vulcanization [14,15].

Recent research has analyzed the effects of graphene nanoplateles (GNP) and organoclay montmorillonite (OMMT) on the strengthening, relaxation and mechanical properties of nanocomposites (NBR/PVC). Thus, it was found that the maximum torque and cure rate increased after adding the nanoparticles, while the same parameters for the OMMT-reinforced nanocomposites decreased. Tensile strength, elastic modulus and hardness were improved by incorporating nanoparticles, while the GNP effect was more intense. A similar trend was observed in the case of NBR samples with higher acrylonitrile (ACN) content [16,17].

Some authors made PVC and NBR composite materials which have in their structure 10%, 20%, 30% and 40% of polyvinyl chloride (PVC). The results obtained showed that a decrease in crosslinking density and heat resistance with an increase in the PVC content was obtained. In contrast, the addition of PVC to NBR resulted in enhancement of hardness, tensile strength, solvent resistance, and filler dispersion [18,19]. 

Additionally, the possibility of recycling PVC and PMMA in the presence of NBR was tried. Thus, the use of PVC/PMMA mixtures is restricted to industrial purposes due to the lower impact performance of the mixture and the lower stability of the PVC phase. Under these conditions, in order to increase the mechanical and thermal performance of PVC/PMMA mixtures, they were efficiently used in a mixture with NBR incorporation, which resulted in improved impact performance and elongation at the break of the recycled mixture. Optimal performances of the mixture were obtained by adding a mass percentage of 9% NBR, Moreover, embedded NBR, determines a stabilization of the PVC phase present in the realized mixture [20,21]. 

A method has been developed for the preparation of thermoplastic vulcanizates of acetyl ethylene vinyl copolymer and natural rubber (EVA/NR TPV) by using peroxide vulcanization. Additionally, these results applied to other thermoplastics that could be crosslinked with peroxide, for example, polyethylene, polystyrene and polyvinyl alcohol. [22]. 

A possible improvement of the properties of composite materials with rubber matrix refers to the realization of nanocomposites based on poly (1,4-cis-isoprene) from Hevea Brasiliensis (natural rubber, NR) and a high surface area nanosized graphite (HSAG) were improved by using HSAG functionalized with 2-(2,5-dimethyl-1H-pyrrol-1-yl) propane-1,3-diol (serinol pyrrole) (HSAG-SP). This type of NR-based nanocomposites indicates that the dispersion of a graphitic material in a rubber matrix can be improved without using an extra-amount of mechanical energy, just by modifying the chemical nature of the graphitic material through a sustainable process, avoiding the traditional complex approach, which implies oxidation to graphite oxide and subsequent partial reduction [23]. Additionally, composite materials made of thermoplastic elastomer with poly(styrene-butadiene-styrene) (SBS) copolymer, which have low costs. Thus, an improvement of the thermal stability of the asphaltene/SBS hybrid materials was obtained [24]. At the same time, composites based on SBS and soy flour were made, which offer special properties [25,26]. The presence of PVC in the composition of acrylonitrile-butadiene rubber reduces the elasticity of molecular chains and, under these conditions, significantly reduces the tensile strength and elongation of the binary mixture. On the other hand, the compressive strength of acrylonitrile-butadiene rubber increases with the addition of PVC. Under these conditions, it has been shown that the deformation rate for rubber is higher after the removal of the applied load, so that when PVC is added, the binary mixture will be subjected to a permanent deformation higher than the rubber. [27,28]. PVC-rubber-type mixtures that aimed to improve the use characteristics of PVC products have been presented in other scientific papers. Thus, composites with a PVC matrix were made by adding rubber: NR, styrene-butadiene rubber (SBR) [29,30]. 

To date, the composite materials shown have not been made using raw materials resulting from waste, such as regenerated rubber. Such materials obtained by mixing rubber with PVC (which come mostly from waste) is a novelty in the field. Additionally, the arrangement of rubber and PVC in layers can be a novelty in the field.

Under these conditions, the research carried out aimed at establishing the conditions under which in the structure of the conveyor belts with rubber matrix can be used raw materials from rubber and PVC waste. Thus, in the paper the following sections were approached: characterization of materials and two types of rubber, PVC and metal reinforcement EP100; establishing the technological parameters used to obtain the conveyor belts by vulcanization; testing at the request of traction of four types of conveyor belts both experimentally and FEM; analysis of changes in materials in conveyor belts produced by accelerated aging; computerized analysis of sample’s material before and after tensile testing; analysis of the obtained results and establishing the conditions for obtaining high-performance conveyor belts.

## 2. Materials and Methods 

The experimental researches aimed at creating conveyor belts that have in their structure materials obtained from the recycling of rubber waste, respectively, PVC. Thus, different rubber recipes were used that have in the structure both reclaimed rubber and rubber powder, obtained by finely crushing rubber waste. The reclaimed rubber was obtained from powder resulting from the crushing of waste tires and conveyor belts. In order to obtain the regenerated rubber, the autoclave devulcanization technology was used. The autoclave was loaded for devulcanization with 900 kg of rubber particles, 7 kg Di-xylene Di-Sulfide (reclaiming agent produced by Arihant oil and Chemicals), and 70 kg of mineral oil (plasticizer and was maintained at a temperature of 225 °C and a pressure of 40 bar for 2.5 h). The particle size was between 0.3 and 0.5 mm, the average of viscosity Mooney ML (1 + 4) 100 °C was 78, and the average elasticity was 17–18. The determination of the elasticity was performed according to ISO 4662/2009 and a Zwick 5109 device was used.

### 2.1. Materials

#### 2.1.1. Rubber Characterization

To perform the experiments, two types of rubber were used, respectively, a rubber denoted R1 for the cover faces of the conveyor belt, and a rubber denoted R2 for the core of the conveyor belts. The two types of rubber (R1, R2) were considered because the conveyor belts must have different properties for the coating faces and to core. Such a conveyor belt was chosen because it must have a very good breaking strength, high flexibility and high reliability. Additionally, the studied conveyor belt is characterized by a width of 2000 mm and a thickness of 18 mm. The compound of the rubber used to make the conveyor belts are presented in Table 1 for the R1 rubber, respectively, for the R2 rubber. Additionally, vulcanizing solutions GDT + 5% Desmodur R type crosslinking agent produced by Covestro AG (Leverkusen, Germany) and trichlorethylene for pickling operations were used. 

Additionally, vulcanizing solutions GDT + 5% Desmodur R type crosslinking agent produced by Covestro AG (Leverkusen, Germany) and trichlorethylene for pickling operations were used.

#### 2.1.2. Characterization of PVC Particles

Regarding PVC, this material was purchased in the form of particles from a company that obtained it from recycled waste. The used flexible PVC particles, whose properties are presented in Table 2, were purchased from the company Crillelmar S.R.L, Targu Jiu, Romania.

The particle size and its distribution is an important property of PVC. The particle size is affected by the type and quantity of suspending agent, reactor geometry and agitation. The size of the PVC particles used had values in the range of 1.5–2 mm. PVC particles were obtained by extrusion.

As for the PVC particles, they were also characterized by the point of view of polydispersion using optical microscopy, and the values obtained were processed using analysis of variance (ANOVA). Thus, the estimation of the average size of PVC particles (average numerical size *D_n_*, average gravimetric size *D_w_*) and the polydispersity index (*D_w_*/*D_n_*) was made by measuring 100 particles.

(1) $$D_{n}=\frac{\sum_{i}N_{i}\cdot D_{i}}{\sum_{i}N_{i}}$$

(2) $$D_{w}=\frac{\sum_{i}N_{i}\cdot D_{i}^{4}}{\sum_{i}N_{i}D_{i}^{3}}$$

(3) $$PDI=\frac{D_{w}}{D_{n}}$$

where *N_i_* represents the number of particles that have the size *D_i_*. 

After measuring the dimensions of the particle and calculating the numerical average size D*_n_*, respectively, the gravimetric mean size *D_w_*, the value of the polydispersity index *PDI* = 0.871 was determined.

#### 2.1.3. Characterization of the Textile Reinforcement

The textile reinforcement used was purchased from Kordárna Plus, Czech Republic and was of the EP 100 type. This type of reinforcement is often used as a reinforcing material for conveyor belts that are subject to low, respectively, moderate stress. The textile reinforcement properties used in the experiments are presented in Table 3.

### 2.2. Methods of Obtaining and Testing Conveyor Belts

#### 2.2.1. Obtaining Conveyor Belts

The research was carried out on a hot vulcanization press of DSLQ conveyor belts, produced by Wagener Schwelm Corporation (Reisholzstraße, Hilden, Germany), which allows the hot vulcanization of rubber conveyor belts with metal cord, with a width of up to 3000 mm. In the case of this type of vulcanization press, the pressure required in the vulcanization process is achieved with the help of hydraulic cylinders. This type of joint presses by vulcanization, of the conveyor belts, has the upper sleepers with hydraulic cylinders, and the lower sleepers and the heating plates are made of special high-quality aluminum, with high resistance to bending and traction, to offer customers more long operation of installations. 

Considering the characteristics of this vulcanization press, it allowed us to adjust some working parameters in a wide range. To make the conveyor belts, the heating was done with the help of vulcanization plates connected to an energy power supply. Additionally, the pressing force was generated with the help of hydraulic sleepers. Initially, a pressure of 80 bar was established. As the temperature of the heating plates increased, the pressure was increased as follows: up to 50 °C—pressure 80 bar; up 80 °C—pressure 100 bar; up to 100 °C—pressure 140 bar; up to 125 °C—pressure 180 bar; up to 145 °C—pressure 200 bar. After the temperature of the heating plates exceeded the value of 100 °C, an increase in pressure was realized up to 200 bar, a parameter that was verified with the help of manometers mounted for this purpose on the installation.

The heating temperature was permanently monitored with the help of thermostats and switching devices. In addition, a thermocouple for temperature monitoring was reinforced in each vulcanization plate. It was also taken into account that the thermometers in the vulcanization plates generally indicate a temperature of about 5–8 °C lower than that on the heating surface. The heating time was calculated from the time of reaching a temperature of 125 °C. The vulcanization time of the conveyor belts was 70 min. After the expiration of the heating time, the process was stopped and the joint was cooled under pressure to a temperature of about 60 °C, in order to avoid the possibility of air bubbles forming inside the strips. Four types of conveyor belts were made from which samples were subsequently taken and subjected to analysis. The conveyor belts were made by vulcanizing the layers of material arranged in the following sequence:

- Samples I: R1—R2—R1—R2—R 1;
- Samples II: R1—R2—PVC—R2—R1;
- Samples III: R1—EP 100—R2—EP 100—R1;
- Samples IV: R1—EP 100—R2—PVC—R2—EP 100—R1.

Sample I was made by arranging layers formed by the two types of rubber, R1 and R2, respectively. Sample II was obtained starting from Sample I’s structure by replacing the middle layer made of R1 rubber with PVC. Regarding Sample III and Sample IV, they also have the reinforcement EP 100 in the structure. Regarding Sample IV, this was modified in relation to Sample III by adding PVC. It was necessary for the rubber foils used to have different thicknesses for the 4 samples to maintain a constant thickness of 18 mm, namely:

- Samples I: the thickness of the foils R1, respectively, R2—3.6 mm;
- Samples II: the thickness of the foils R1, respectively, R2—4 mm;
- Samples III: the thickness of the foils R1, respectively, R2—5.6 mm;
- Samples IV: the thickness of the foils R1, respectively, R2—3.7 mm.

**Figure 1:**
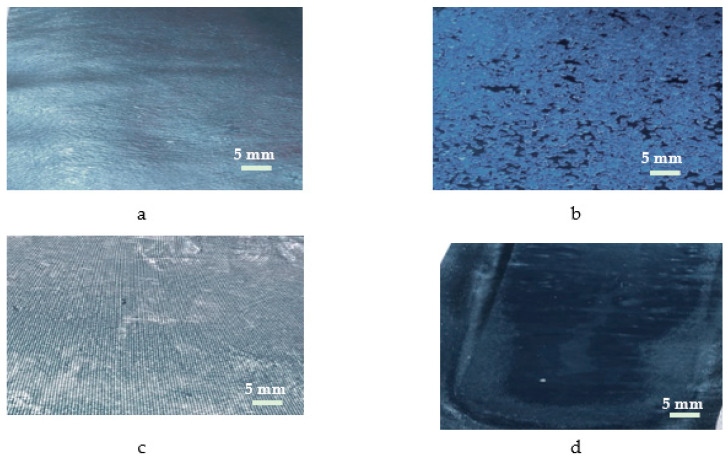
Stages of the process of obtaining conveyor belts. (**a**) arrangement of rubber foils; (**b**) arrangement of PVC particles; (**c**) arrangement of the textile reinforcement EP 100 and the trichlorethylene stripper; (**d**) the conveyor belt obtained.

An image of how to obtain conveyor belts is shown in Figure 1.

#### 2.2.2. Methods for Testing the Strips in Terms of Mechanical Properties

##### Experimental Testing of Samples to Tensile

Conveyor belts were tested at traction and these tests were performed in accordance with the SR ISO 37/2010 standard. The tensile test machine used was a mechanical test machine of the type Instron 558). The machine is equipped with the Bluehill 2 software used to command and control the machine but also to process the results. The Bluehill 2 program allows the following actions to be performed: automatic calibration of the sensors; generating predefined and user-edited reports; system monitoring; viewing results in real time; the possibility of determining conventional and real characteristic curves and plasticity characteristics.

The test method was developed in the own software of the Instron type test machine, namely Bluehill 2. Regarding the test speed, it was set at 500 mm/min. The samples had a width b_0_ = 30 mm and a length l_0_ = 250 mm, and the free measuring distance l = 200 mm. Prior to the experiments, the samples were kept in the laboratory at a constant temperature of 25 °C.

The output collected data were: maximum force [N] and elongation corresponding to maximum force [mm]. The two output dates were chosen at the expense of the breaking force and the elongation at break because the moment when the test machine detects the break is when the tensile force suddenly decreases by 10–20%.

##### Analysis of the Tensile Behavior of the Samples through FEM

The analyses in the present study were explicit dynamical analyses and were run using Ls-Dyna. In Ls-Dyna, the composite materials can be defined in three ways: microscale, mesoscale single-ply or mesoscale laminate. The microscale modeling consists in the modeling of the entire model at material level (practically, each 3D fiber, the matrix in which the fiber is embedded, and the interface between them are modeled) and it is used only for micromechanical elements because it conducts to a very large number of finite elements and, obviously, to extremely big analysis durations. The mesoscale single-ply modeling is used when we have a material made of only one type of cell or more types of cells that occur repeatedly. It is used for heterogenic materials. This type of modeling is generally used at the numerical simulation of textile materials. A 3D mosaic model approach was developed to predict composite behavior at the mesoscale by Olsson [31].

The mesoscale laminate modeling is used to model the composite materials composed of more layers, exactly as it is the case of the current paper. This type of modeling is the most frequently used because it does not require an increase in the number of nodes and an increase in the analysis duration [32]. At this type of simulation, the failure of the elements can occur either inside the structures of the composite material (interlaminate) or by the unbinding of the layers (delaminate). Additionally, if we are referring to the interlaminate failure, Ls-Dyna can account for the fracture of the bonding piles.

For the simulation of the interlaminate behavior, we can use thin shell elements (that have a one-layer structure) that allow for a 2D stress state, thick shell elements (that have a solid element, multilayer structure) that allow for 2D and 3D stress states and solid elements (with a structure similar to thick shell elements) that allow for a 3D stress state. Both shell elements have a large number of integration points, while solid elements only have one integration point. For the interlaminate behavior, cohesion elements are used.

The physical modeling possibilities for laminar composite materials, as in the present paper, are the use of a single type of shell element, the use of layers for solid elements and the use of layers for shell elements. From the beginning, we have eliminated the possibility of using a single shell element because this type of modeling, although presenting the advantage of a shorter duration for the analysis, does not allow the simulation of delamination. Delamination is a critical failure mechanism and it is caused by high interlaminar stresses towards low through-thickness stress. The delamination can have multiple causes, but in the present situation, it can occur due to the presence of straight free edges, situated at the border of the sample. By using layers of solid elements and layers of shell elements, the bonding between the layers can be done either by using a tiebreak type of contact or by the physical modeling of the adherent layer and use of cohesive elements (with a 0 or a nonzero declared thickness) and cohesive-type materials. The tiebreak contact is essentially a surface-to-surface contact.

#### 2.2.3. Methods for Testing Conveyor Belts in Terms of Thermal Shock Behavior

Conveyor belts can work in special conditions, especially in terms of temperature conditions. In this sense, the most common stress conditions consist of temperature cycles between −40 °C and 85 °C. Thus, environmental conditions have a major influence on the functionality and reliability of conveyor belts. To detect latent weaknesses in the shortest possible time, a typical temperature test is often insufficient. Thus, the samples were subjected to several sudden changes in temperature. Under these conditions, the elastic properties of the conveyor belts were determined, both immediately after manufacture and after the application of thermal shocks.

A Zwick/Roell Z05 TN device with testXpertII software version 3.6 produced by BRECON Vibrationstechnik GmbH Stolberger, Cologne, Germany was used for conveyor belt elasticity measurements. This type of device has a wide range of equipment options that allow the use of zwickiLine for testing plastics, elastomers, metals, composites, paper, cardboard, textiles, foams, food and components. 

A thermal chamber was used for the thermal aging of the conveyor belts—Temp Shock test chamber Votsch VT3 7012 S2 produced by Test Equipment Co., Ltd. Jin Hui Industrial Park, China. This thermal chamber allows very fast temperature changes in the range of −80 °C–+220 °C. This way of testing conveyor belts allows a reduction in the occurrence of the incidence of early failures and the improvement of the manufacturing conditions of conveyor belts to increase their reliability.

Regarding the thermal aging application regime, it was established taking into account the following parameters:

- A total of 2000 cycles of variation between −40 °C and 85 °C;
- The times to accommodate varied, at the first 700 cycles it was 45 min, and for the rest, it was 15 min.

Establishing an accommodation time of 15 min. for the last 1300 load cycles was considered sufficient given the small mass and relatively large surface area of the samples. The accommodation was made under forced conversion (ventilation). 

The adoption of a temperature variation between −40 °C and 85 °C was made taking into account the real conditions in which the conveyor belts can be requested. Regarding the penetrator used in the testing process, it was one with a spherical head with a diameter of 7.96 mm and a radius at the tip of 3.98 mm.

#### 2.2.4. Analysis of the Materials Structure from Samples

The samples have several materials in the structure, being obtained a series of composite materials with rubber matrix, respectively, PVC additions and textile reinforcement. In order to know as well as possible the correlation between the structure of the composite material and its physical-mechanical properties, in the researches, the aim was to establish the homogeneity of the structure of the composite materials and to identify the possible inhomogeneities in them. Thus, the research tested materials using radiation. This method of nondestructive testing was chosen because it offers the best results for these types of material.

An equipment 3D X-ray Measurements for Quality Assurance ZEISS METROTOM was used to test the materials. This generation of the ZEISS METROTOM 1500 CT scanner provides the best proof that advanced X-ray technology is reliable and offers the best results in material structure analysis. With a computed tomography system from ZEISS, a measurement and inspection can be successfully performed by X-ray scanning of materials providing very important information on the structure of materials. This third generation of the tomograph computer has in the structure a new 3k detector that generates sets of 3D volume data with higher resolution, i.e., more voxels that allow the detection of much smaller defects. VGSTUDIO MAX software was used to process the images resulting from the material testing. This software inspection solution has six modules and is designed for industrial product inspection. Additionally, this software solution stands out for its higher computing speed (GPU computing), which drastically shortens the time expected for results. At the same time, the extensive use of 3D through the GUI makes Digi Inspect easy to use.

## 3. Results and Discussions

The addition of PVC in the polymeric structure of the conveyor belts determines significant changes in the morphology of the composite material and of the macromolecular mobility in the delimitation layers between rubber and PVC. The new microstructure of the conveyor belts determines their different behavior at the tensile stress, but also from the point of view of preserving their properties after the application of an artificial aging process by thermal shocks.

### 3.1. Determining the Behavior of Conveyor Belts to Traction

The analysis of the behavior to traction for the conveyor belts is very important because in operation this type of products are subjected primarily to this type of stress. The influence of the addition of PVC on the traction behavior of the conveyor belts both without and with textile reinforcement was especially analyzed. 

Tensile testing was performed using both FEM and experimental. The use of layers and shell elements presents a disadvantage in the current situation, because at these elements the through-thickness component is set to zero. The solid elements, although requiring larger analysis durations, have the advantage of having a corresponding through-thickness stress component that allows the determination of the interlaminate shear stress.

**Figure 2:**
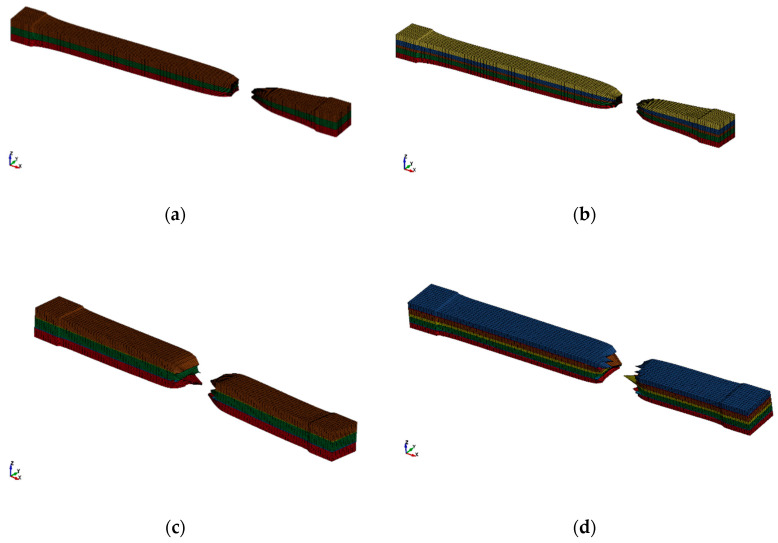
The way to break the samples subject to tensile following the simulation with FEM. (**a**) SI sample; (**b**) SII sample; (**c**) SIII sample; (**d**) SIV sample.

Based on the previously mentioned, we have modeled the four types of samples using the PrePost Ls-Dyna processor. The samples were then used for the tensile test. The finite elements used were solid elements, as can be noticed in Figure 2. The samples were tested until the failure occurred. Thus, it is observed that the deformations before breaking are higher in the case of the SI sample, Figure 2a, in relation to SII sample, this being explained by the presence of PVC in the composition, Figure 2b. Additionally, by comparing the deformations before breaking suffered by the SIII sample, Figure 2c, in comparison with the SIV sample, Figure 2d, it is observed that the deformations suffered in the case of the SIV sample are smaller, which can be explained by the presence of PVC.

**Figure 3:**
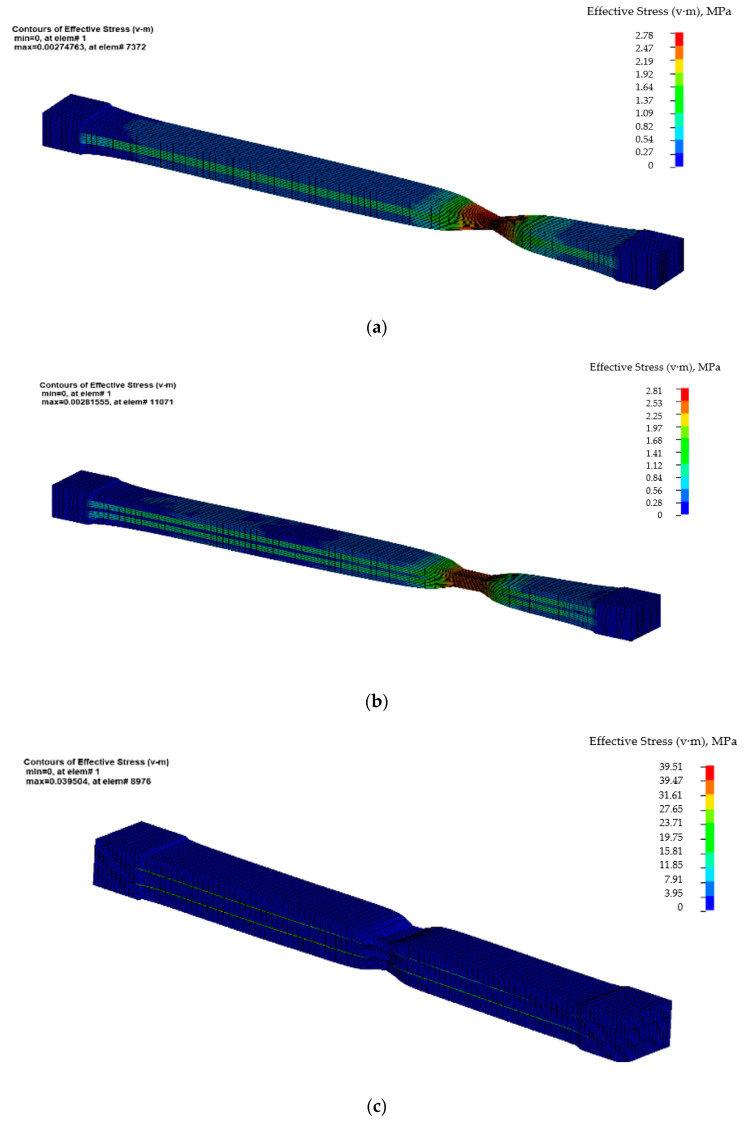

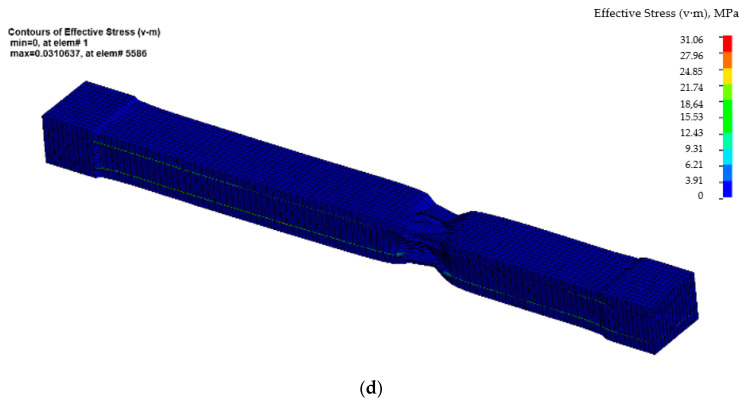
The equivalent von Mises stress for the samples before failure. (**a**) SI sample; (**b**) SII sample; (**c**) SIII sample; (**d**) SIV sample.

Figure 3 shows the four samples, immediately after the failure. The materials used in the analysis were MAT_SIMPLIFIED_RUBBER_WITH_DAMAGE, which is material number 183 in Ls-Dyna, for all the types of rubber used in the analyses, respectively, MAT_PIECEWISE_LINEAR_PLASTICITY, which is material number 24 in Ls-Dyna, for the EP100 reinforcement. For the MAT_SIMPLIFIED_RUBBER_WITH_DAMAGE, the input parameter was the force-displacement curve resulted from the tensile test, and for the MAT_PIECEWISE_LINEAR_PLASTICITY, the true stress-true strain curve. The contact between the laminated layers was a TIEBREAK_SURFACE_TO_SURFACE contact. The results were focused on determining the equivalent von Mises stress and determining the force-displacement curve, which was compared to the experimentally obtained one.

In Figure 3, the resulting equivalent von Mises stress is presented. By analyzing these figures, it can be noticed that, for the first two sets of samples, made of only rubber and PVC, the value of tensile stress before failure is small, but compensated by a larger tensile strain. For the second set of samples, the stress before failure increases up to 39.51 MPa for the SIII sample, but the tensile strain decreases. For the SIV sample, the maximum value of the equivalent von Mises stress reaches 31.06 MPa. The corresponding values of the equivalent von Mises stress for SI sample and SII sample are 2.75 MPa, respectively, 2.82 MPa. Anyway, the maximum value of the von Mises stress occurs in the EP100 reinforcement for both SIII and SIV samples. 

**Figure 4:**
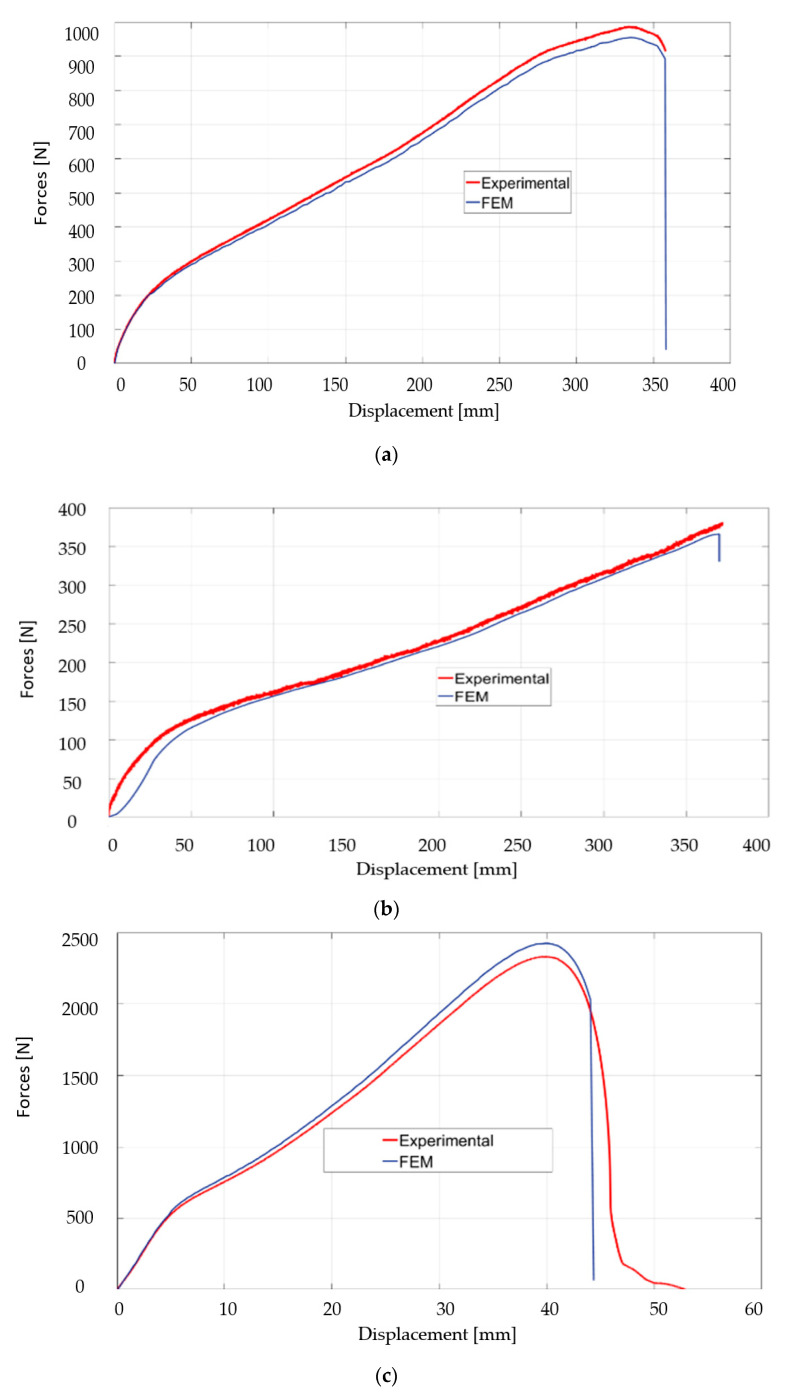

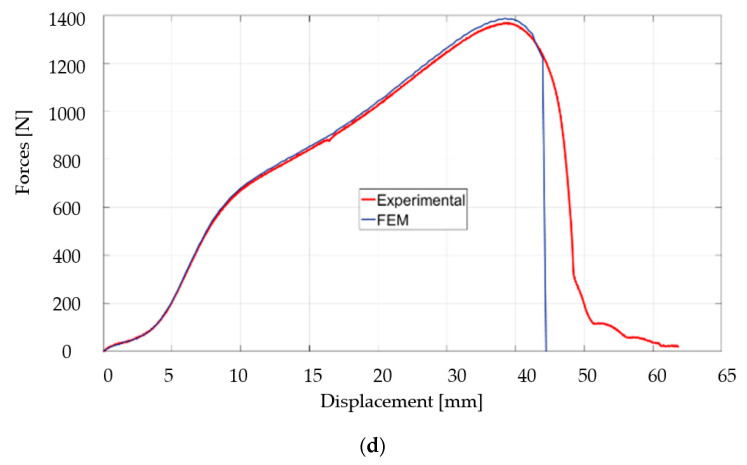
The dependence of tensile stress according to tensile strain. (**a**) SI sample; (**b**) SII sample; (**c**) SIII sample; (**d**) SIV sample.

Following the analysis of the above-presented figures, it can be observed that the adherence of the layers is maintained over the course of the test, and that the delamination of the layers does not occur. The results of the experimental test and FEM for the four samples are presented in Table 4. The force-displacement curves for the four samples obtained both experimentally and by FEM are presented in Figure 4.

**Figure 5:**
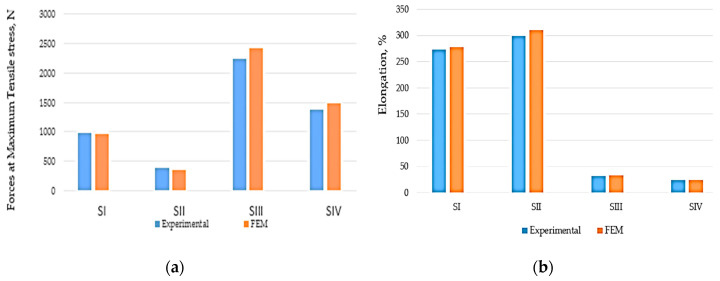
The evolution of the mechanical properties of composite materials. (**a**) Forces at maximum Tensile stress, (**b**) elongation.

The graphical analysis of the mechanical properties of the material from the four samples was presented in Figure 5.

From the analysis of the results presented in Table 4, but also from the graphical evolutions presented in Figure 4 and Figure 5 it is observed that both the experimental research and the FEM allow obtaining very close results for the four samples. Thus, the FEM method can be used in an optimal way, subject to the imposition of appropriate conditions.

From the analysis of the obtained results, a different behavior to traction was observed for the four samples. Thus, maximum forces and tensile stress were obtained for the SIII test piece, which is composed of the two types of rubber, but also by the EP100 reinforcement. This fact influences the elongation, and thus the most value is obtained in the case of the SIII test tube. This tensile behavior can be explained by the addition of PVC particles, but also by the presence of EP 100 reinforcement in the structure of certain specimens. Additionally, the equivalent of von Mises stress has a considerable value if PVC is added to the samples, which proves that this technical solution allows obtaining composite material with high mechanical properties.

Thus, it was interesting to analyze the traction behavior by comparing the results obtained for the SI and SII and SIII and SIV samples. This comparison was necessary because Sample SI has only rubber in the structure, and in the case of Sample SII, PVC was added. As for Sample SIII, it has in structure, in addition to rubber, textile reinforcement (EP100), and Sample SIV has a structure similar to test tube Sample SIII, but to which PVC particles have been added. The analysis of the samples to the tensile provides us with information on the way in which the conveyor belts could behave in operation. The addition of PVC causes a decrease in the tensile stress of the samples and this can be observed both in the case of Sample SII and in the case of Sample IV. This can be explained by the fact that the presence of PVC in the structure of the conveyor belts can cause a reduction in the possibilities of making the connections inside the rubber during the vulcanization process. A special situation appeared in the case of Sample SII which contains rubber and PVC, in the sense that, although a very high value of foreign tensions was registered, it did not break. Additionally, the maximum tensile stress had a rather low value in the case of Sample II compared to Test Tube I, namely about five times lower.

In the case of samples SIII and SIV, substantial differences were observed in terms of stress and tensile strain, but smaller than the situation in the case of Samples I and II, respectively, and this can be explained by the fact that the presence of the textile reinforcement causes a change of conveyor belt properties. Additionally, from Figure 4c,d, respectively, it can be observed that the three samples that have in the structure only rubber and textile reinforcement show a doubling of the value of the stress tensions in comparison with Sample IV, which contains PVC. It should also be noted that the foreign tension has a value, at the moment that breakage occurs samples, of approximately 0.2 mm/mm in the case of the SIII sample and 0.27 mm in the case of the SIV sample. It should be noted that the difference between the value of the foreign tensile from the beginning of the rupture and the moment when the final rupture occurs (tensile stress = 0) had a value of 0.195 mm/mm in the case of SIII and 0.7 mm/mm in the case of the Sample SIV, which proves once again that PVC-containing samples have better elasticity, and their rupture occurs over a longer period of time, which offers a great advantage for conveyor belts that are not strongly demanded, but to which a high elasticity is required.

From the analysis of the results obtained after the tensile test, it can be concluded that the presence of PVC in the structure of the conveyor belts determines a substantial reduction of the tensile stress, but also an increase in its tensile strain values. Thus, by adding PVC, a conveyor belt structure can be obtained that has appropriate properties depending on the field of use, which was possible until present only by modifying the rubber structure by adding natural rubber which has a very big cost price. At the same time, it has been shown that the addition of SiO_2_ in the structure of membranes containing natural rubber and PVC can cause an increase in the tensile strength of conveyor belts [24]. Thus, the structure of a conveyor belt can be optimized in terms of PVC and SiO_2_ content.

### 3.2. Determining the Elasticity of the Materials in the Conveyor Belts Subjected to the Accelerated Aging Process

The accelerated aging process of the materials from the analyzed conveyor belts was performed in compliance with the SR ISO 188:2010 standard. The realized the accelerated aging tests are designed to assess the relative resistance of rubber or plastics to damage over time. For this purpose, the tested materials must be subjected to controlled deterioration conditions, for determined periods of time, after which the respective characteristics are measured and compared with the initial properties of the materials. In the case of accelerated aging, the materials are subjected to a test environment designed to produce the effect of natural aging in a shorter time. In the case of materials such as rubber and PVC, it is recommended to measure the deterioration through changes in the elasticity property which has special practical importance. Thus, the environmental conditions have a major influence on the elasticity properties of the rubber, respective PVC, and this influences the functionality and reliability of the parts made of such materials as is the case of the conveyor belts analyzed.

**Figure 6:**
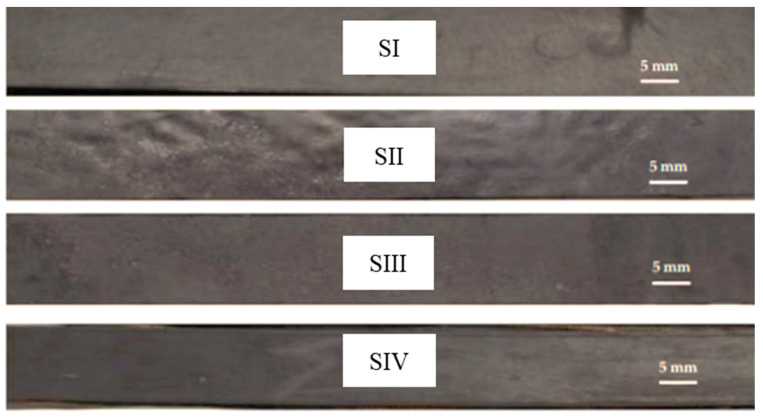
The shape of the samples used in the accelerated aging process.

The test samples were taken from the four types of conveyor belts analyzed and have had the following dimensions: length L = 250 mm, width l = 30 mm, thickness b = 18 mm. An image of the samples subjected to the accelerated aging process is presented in Figure 6. Figure 6 shows the shape of the outer surface of the samples made. Thus, in the case of the SII specimen, the outer surface has some cavities, determined by the presence of PVC, which is no longer valid in the case of the SIV sample, due to the fact that PVC particles are prevented from migrating to the outer surface by the presence of EP100 reinforcement.

The process of testing the elasticity of the materials in the conveyor belts was carried out under the same conditions, both in the case of new belts and those subject to the accelerated aging process. In this sense, several stress cycles were performed on the samples, which took place with the same pressing speed of 30 mm/min, but with different values of the pressing force. In this sense, the pressing force gradually increased from an initial value of 10 N to a final value of 200 N as follows: Stage 1—F = 10 N; Step 2—F = 20 N; Step 3—F = 50 N; Step 4—F = 70 N; Step 5—F = 100 N; Step 6—F = 150 N; Step 7—F = 200 N.

**Figure 7:**
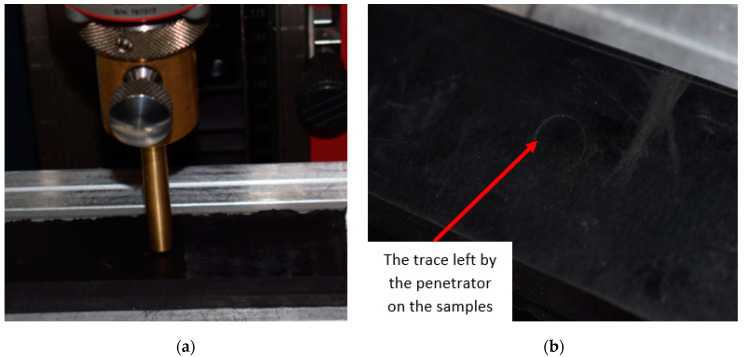
Scheme to artificial aging testing of samples. (**a**) the initial phase of the testing process; (**b**) the final phase of the testing process and the trace left by the penetrator on the test piece.

By pressing the penetrator on the surface of each sample, a trace of circular form remained, considering the geometry of the penetrator used in the testing process, Figure 7.

**Figure 8:**
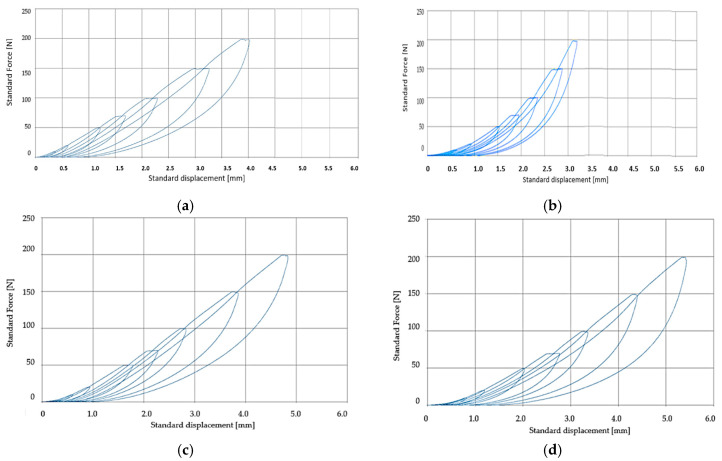
Standard displacement obtained for the different forces applied for initial samples. (**a**) SI sample; (**b**) SII sample; (**c**) SIII sample; (**d**) SIV sample.

**Figure 9:**
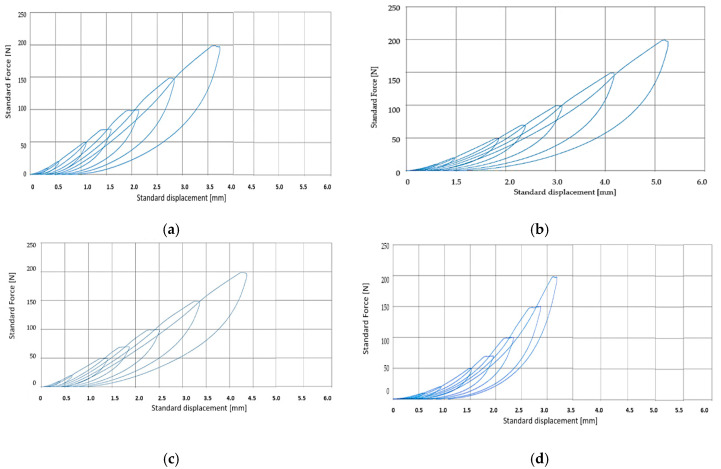
Standard displacement obtained for the different forces applied for accelerated aging samples. (**a**) SI sample; (**b**) SII sample; (**c**) SIII sample; (**d**) SIV sample.

Regarding the graphical evolution of standard displacement, obtained for the different forces applied, it is presented in Figure 8 for initial samples and in Figure 9 for accelerated aging samples. Additionally, the values obtained for standard displacement for the four samples, both in the initial state and after the application of accelerated aging, these are presented in Table 5.

The analysis of the results obtained in the elasticity testing process for the 4 types of samples showed that the presence of PVC, but also of the EP100 textile reinforcement, have an influence on the accelerated aging process of the materials from the obtained conveyor belts. Thus, from the analysis of the accelerated aging process of the SI sample, a decrease of the elasticity of the material was found after the application of the aging process, which is a normal thing for the products that have only rubber in the structure. However, the largest difference in elasticity was observed when forces of 150 N and 200 N were applied, respectively.

Regarding the SII sample, it has PVC in structure, and the results of the tests performed showed that the elasticity of such a conveyor belt increases with the application of accelerated aging treatment. This is a result that can be a novelty because, usually, the elasticity had to decrease. This result can be justified by the fact that the presence of PVC in the middle of the conveyor belt can cause the penetration of PVC particles into the rubber layers before it changes its properties by vulcanization. Thus, a composite with a complex structure can be obtained and not a lamellar one. The obtained result demonstrates the fact that if it is desired to obtain some conveyor belts with high resistance to the aging process, it is possible by distributing some PVC particles in the rubber matrix. It should also be borne in mind that PVC has a relatively low coefficient of friction which can be improved by dispersing silicon carbide (SiC) on its surface [31]. 

Regarding the SIII and SIV samples, it was observed that the presence of the textile reinforcement in the structure substantially modifies the behavior of the material in the conveyor belts in terms of accelerated aging behavior. Thus, in the case of SIII samples, the differences in elasticity between the initial samples and those subjected to the accelerated aging process are larger than the SI sample, which did not contain textile reinforcements. At the same time, the presence of PVC in the structure of the SIV sample does not determine a very different behavior in relation to the SIII sample, which does not have in the PVC structure, but also in this situation, the presence of PVC determines a better elasticity of the conveyor belt, before and after the accelerated aging process.

### 3.3. Determining the Structure of Samples Materials

**Figure 10:**
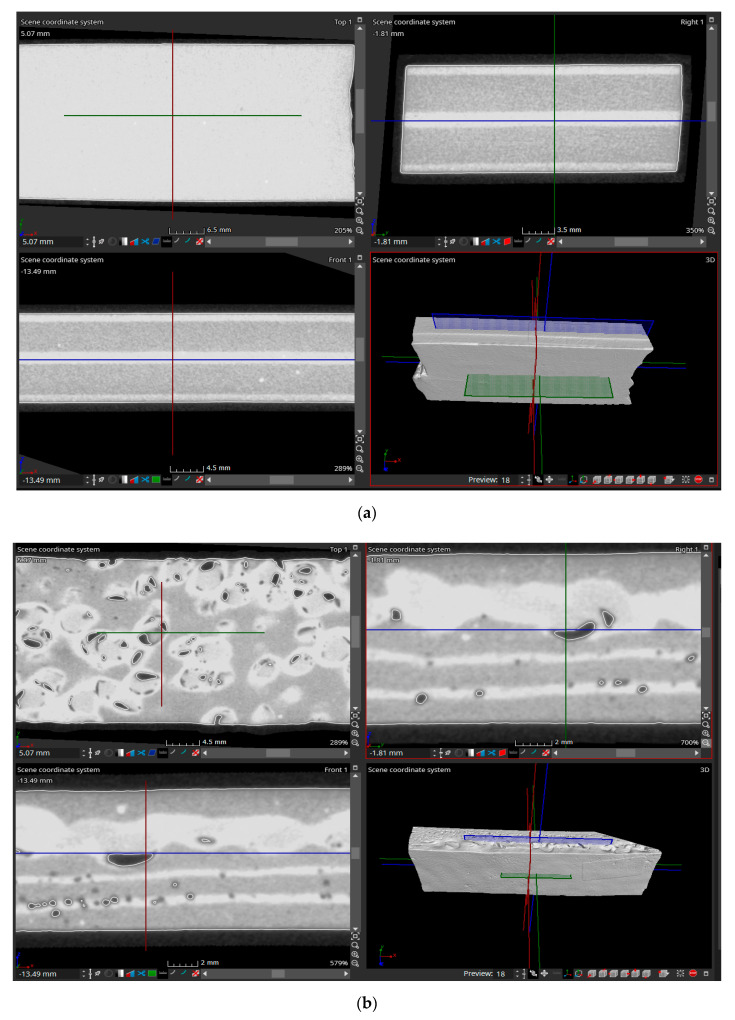

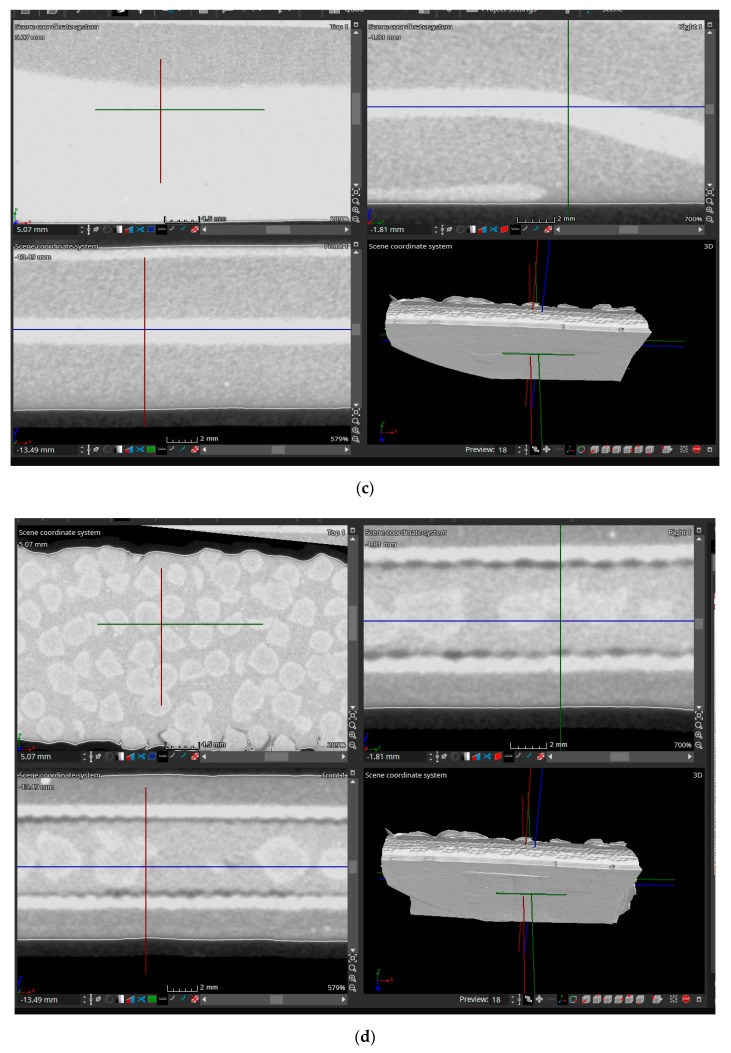
The structure of the material from the initial samples unsolicited to traction. (**a**) SI sample; (**b**) SII sample; (**c**) SIII sample; (**d**) SIV sample.

**Figure 11:**
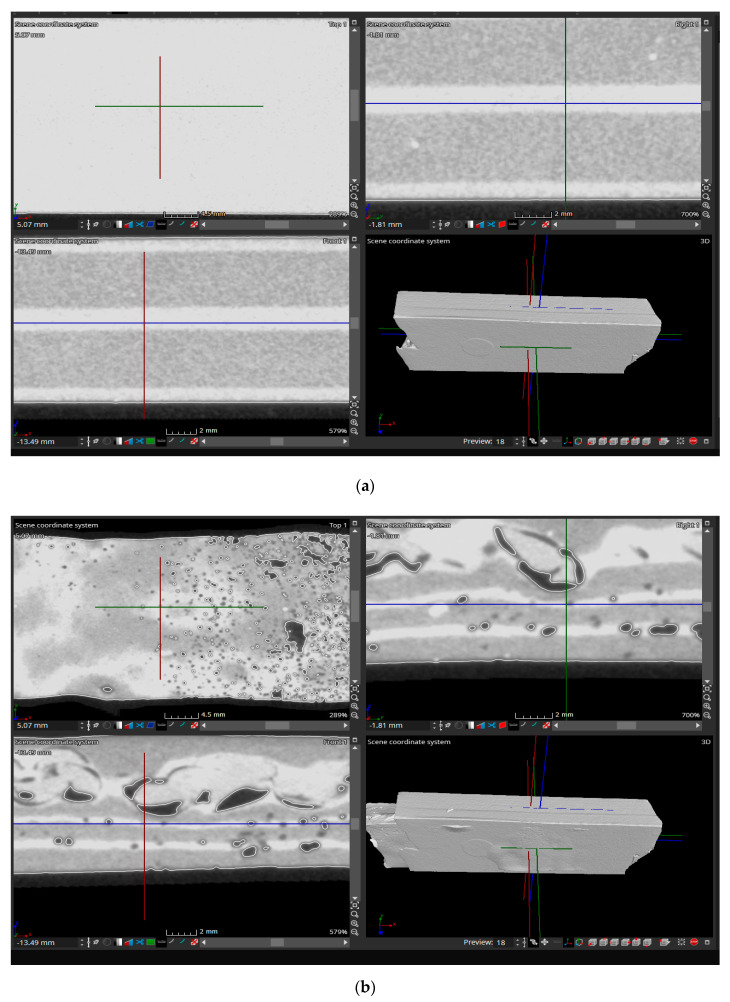

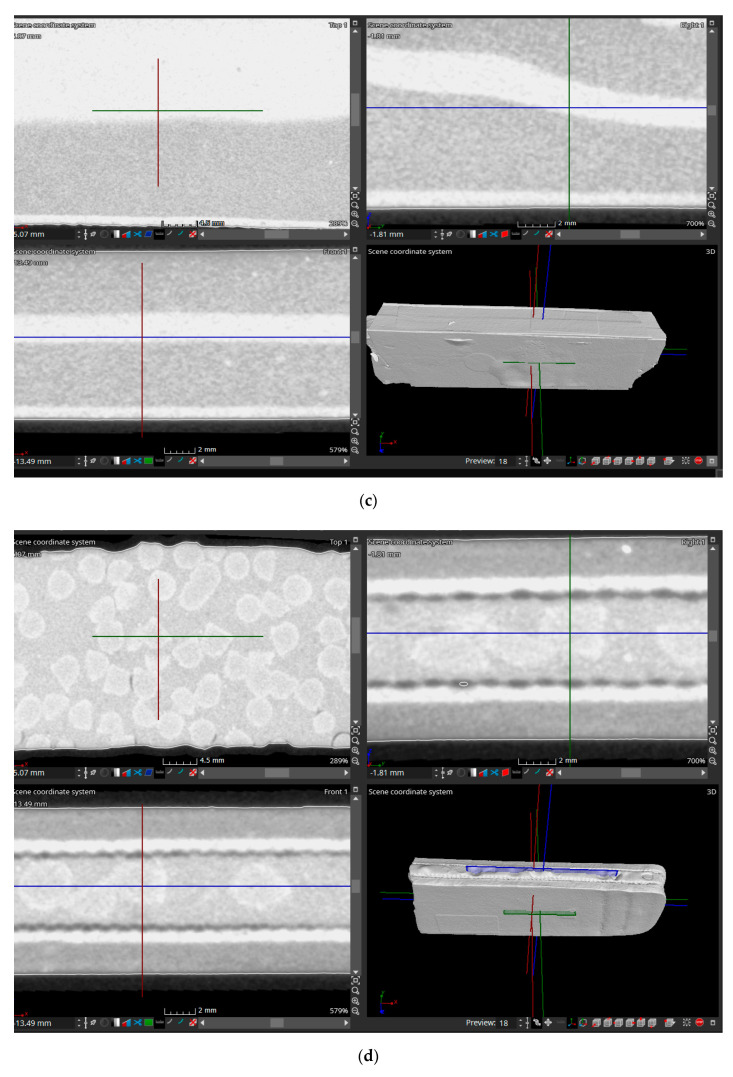
The structure of the material from the initial samples solicited to traction. (**a**) SI sample; (**b**) SII sample; (**c**) SIII sample; (**d**) SIV sample.

In order to determine the structure of the test piece materials, an analysis of them in the initial state and after the tensile stress was taken into account. This type of analysis establishes the way in which the connections made during the vulcanization process of the rubber are broken or preserved in the sense that the defects (gaps in the test material) can be observed very well. The 4 samples (SI, SII, SIII, SIV) were subjected to scanning both in the initial state (before the tensile stress) and after the tensile stress. The structure of the material obtained after the X-ray scan is shown in Figure 10 for the unsolicited samples, and the structure of the samples required for traction is shown in Figure 11.

The scanning of the structure of the test tube materials with the help of X-rays allowed the establishment of conditions related to the composition of the materials, but also to the technologies that must be used so as to obtain materials that have high performance in operation. The scan allowed the observation of the structure of the samples in three different planes. Thus, the used software allowed the movement of the three planes so that the structure of the material could be observed in many sections. Figure 10 and Figure 11 show, in the upper left, a section through the sample in a vertical plane in a longitudinal direction, and in the upper right, with a vertical plane that cuts the sample in a transverse direction. In the lower-left is presented the section obtained by cutting the sample with a horizontal plane in a longitudinal direction.

The structure of the material in the SI samples is presented in Figure 10a, for the initial unsolicited state of traction, respectively, in Figure 11a, after the tensile stress. From the analysis performed, it was found that following the tensile stress, a series of gaps appear in the material, the largest being in the area where the R2 type tire exists. The presence of larger gaps in this type of material can be explained by the fact that the composition of R2 type rubber includes less natural rubber than R1 rubber, which influences its behavior at the tensile stress, having a lower elasticity.

PVC particles were introduced in the structure of the SII samples in the central area and, thus, it was possible to establish the influences that the addition of such a material has on the properties of this type of composite material. The analysis of the structure of the material from the SII samples is presented in Figure 10b before the tensile stress and in Figure 11b after the tensile stress. Both Figure 10b and Figure 11b show that, although PVC particles were introduced only in the central area of the sample, they are present in its entire thickness. This can be explained by the fact that the particles entered the rubber structure before the vulcanization process started due to the pressing force generated by the vulcanization installation. It was also found that the best adhesion between the PVC particles and the rubber was achieved in the area of the R2 type rubber. This is highlighted by the gaps between the rubber and the PVC particles both before and after the tensile stress. The difference in adhesion between the PVC particles and the two types of rubber can be explained both by their different chemical composition and by the different temperatures at which the vulcanization process begins for rubbers R1 and R2, respectively.

Regarding the influence of the presence of the textile reinforcement EP 100 on the homogeneity of the structure of the materials, it was found that, in the case of Sample SIII (Figure 10c and Figure 11c), which has in the structure only rubber R1 and R2, the presence of the textile reinforcement influences the structure of the material after applying the tensile stress, in the sense that the size of the rubber gaps is very small, and the textile reinforcement changes its thickness upon stress due to the breaking of the threads in it.

Regarding SIV, it has the most complex structure, being formed both by the two types of rubber and by PVC particles, respectively, textile reinforcement EP 100. From the analysis of the structures presented in Figure 10d and Figure 11d it is observed the fact that the presence of the textile reinforcement prevents the movement of PVC particles on the entire thickness of the test piece. This movement is restricted by the presence of the textile reinforcement. Thus, the PVC particles penetrated only in the R2 type rubber, which allowed obtaining a high adhesion with it, and this was also demonstrated in the case of the SII test. This test tube structure is a high-performance one, without gaps in the material neither before nor after the tensile stress, being an optimal solution for the realization of high-performance composite materials with rubber matrix and PVC additions.

Thus, the carried analysis showed that the presence of PVC in the structure of the conveyor belts causes an increase in their elasticity, and to avoid loss of elasticity in the aging process it is recommended that the conveyor belts be made in a structure in which the textile reinforcement do not be interposed between the PVC and the superficial rubber layers of the conveyor belt.

A possible solution to improve the vulcanization conditions of the conveyor belts that have in the PVC structure would be the use of ultrasound that would allow a better uniformity of the parameters of the vulcanization process (temperature, pressure) in the whole mass of material of conveyor belt [33,34,35]. 

The obtained results confirm that there may be good compatibility between PVC and silicone rubber, which allowed the improvement of stress-strain behavior [29,36,37]. Additionally, the obtained results showed that products made of rubber with PVC additives have a better behavior in the aging process that can be caused by heat or UV radiation. At the same time, these results can be explained by the fact that it is possible that rubber vinyl groups could be the reason for maintaining the mechanical properties unaltered in the conditions of undergoing the aging process of these products. The effects determined by the addition of PVC in the rubber matrix can also have effects in the sense of reducing the percentage of water adsorption [38] which, in the case of products, such as conveyor belts working in special environmental conditions, has special importance.

Recent studies have shown that PVC/NBR (nitrile-butadiene rubber) mixtures can be used to obtain fuels-resistant products such as biodiesel. However, these mixtures have a complex formula that includes: NBR/PVC, conductive carbon black; rice husk ash containing at least 90% SiO2; synthetic amorphous silica; graphene oxides involving high costs [39]. The solution proposed in the research offers the possibility to obtain a rubber/PVC mixture that has a formula that includes reclaimed rubber and PVC particles obtained from waste, thus being able to obtain much cheaper products. 

Additionally, research was conducted dedicated to the study of the kinetics of the cure reaction of rubber nanocomposites based on the mixture (NBR/PVC) (70/30), reinforced with graphene. Two series of nanocomposites were prepared using two grades of NBR [33 and 45% of acrylonitrile (ACN)] with a general-purpose PVC [40,41]. However, this study only analyzes the influence of graphene content on activation energy. Instead, the results obtained in our research provide a more detailed picture of the influence of PVC on several properties of the tested products, and all these represent news in the field.

A very important thing analyzed in the research refers to the fact that the samples were obtained by depositing layers of rubber namely of PVC particles, which was not addressed in previous research when the rubber/PVC compositions were obtained by mixing the two components [16,22,25,40]. By adopting this method of obtaining the specimens, conditions have been created to establish the type of rubber (R2) to which the PVC particles adhere best.

Under these conditions, the research carried out brings an important scientific contribution in the field in the sense that the tests performed were performed on real products that can be used in practice and demonstrate the technical advantage represented by the presence of PVC in such products.

## 4. Conclusions

The obtained results demonstrated that the addition of PVC in the structure of rubber conveyor belts is a solution for obtaining superior performance in the operation of such products.

Following the stretching request to which the conveyor belts with PVC structure were subjected, the following were established: the addition of PVC in the structure of the conveyor belts determines a reduction of the tensile stress and this can be explained by the fact that the realization of the connections from the rubber matrix that is made during the vulcanization process is prevented by the presence of the PVC particles; a decrease in tensile stress was also observed in the case of conveyor belts containing rubber, PVC and textile reinforcement, but this decrease is much reduced compared to conveyor belts not containing textile reinforcement; it was found at the same time that, in the case of conveyor belts containing PVC, a substantial increase in tensile strain is obtained and, in these conditions, it can be said that these conveyor belts have much better elasticity, accepting very large deformations until the appearance of break; the use of PVC in the structure of the conveyor belts represents a solution for the optimization of their properties so that they have superior performances adaptable to the exploitation conditions; testing the conveyor belts in terms of preserving the properties of elasticity following an accelerated aging process has shown that the presence of PVC in the structure of conveyor belts that have rubber and PVC structure determines a preservation and even an increase in elasticity following the application of aging expedited; if in the structure of the bands, besides rubber and PVC, textile reinforcement on is used, a reduction of elasticity was found due to the accelerated aging process, and this is determined by the fact that the presence of the textile reinforcement prevents the connection between PVC and rubber from the surface layer; the use of the FEM method represents a solution for the analysis of the tensile behavior of these types of material, subject to the correct establishment of the conditions at limit; the analysis of the material structure from samples showed that a better adhesion between rubber and PVC is found if PVC is incorporated in the type of rubber R1; the use of PVC in the structure of the conveyor belts with rubber matrix represents a solution for the realization of conveyor belts that are not subjected to high mechanical stresses but that work in environments where the rubber is subjected to an accelerated aging process.

Future research will aim to optimize the structure of rubber matrix conveyor belts, which have PVC in the structure, so that conveyor belts with a range of mechanical properties can be obtained that can withstand working environments that cause accelerated aging of rubber. 

## Figures and Tables

**Table 1:** Rubber compound.

Materials	The Rubber Compound, phr	
	R1	R2
Natural rubber (Standard Indonesia Rubber-20)	30	11
Styrene-butadiene synthetic rubber (SBR-1723 TDAE)	10	29
Poly-butadiene synthetic rubber SKD ND (Nizhnekamsk Russia)	25	25
Reclaimed rubber (ARTEGO Romania)	20	20
Rubber powder with size 100 µm (ARTEGO, Romania)	15	15
Naphtha	7	6
Carbon black HAF 330	8	8
Antioxidant 4010NA/LG(IPPD) (HENAN, GO BIOTECH, China)	3	3
Stearin	5	6
Vulcanization accelerator DPG (standardQ/CNPC55-2001)	4	4
Sulfur	3	3
Total	130	130

**Table 2:** Properties of flexible PVC particles.

**Flexible** **PVC**	**Properties**				
	**Density, g/cm^3^**	**Thermal Conductivity** **W/(m·K)**	**Yield Strength** **MPa**	**Resistivity** **Ω** **·m**	**Surface Resistivity** **Ω**
	1.1–1.35	0.14–0.17	10–24.8	10^12^–10^15^	10^11^–10^12^

**Table 3:** Textile reinforcement properties (Kordárna Plus, Czech Republic).

**EP 100**	**Material**		**Strength, N/mm**		**Elongation, %**		**Area Weight,** **g/m^2^**	**Thickness,** **mm**	**Adhesion,** **N/mm**
	**warp**	**weft**	**warp**	**weft**	**warp**	**weft**	**-**	**-**	**-**
	PES	PA 66	min. 120	min. 60	23 ± 5	30 ± 5	350 ± 25	0.60 ± 0.15	min. 10

**Table 4:** The results of the tensile tests.

Samples	Forces at Maximum Tensile Stress, N (FEM)	Equivalent von Mises Stress, MPa (FEM)	Elongation, % (FEM)	Forces at Maximum Tensile Stress, N (Experimental)	Equivalent von Mises Stress, MPa (Experimental)	Elongation, % (Experimental)
SI	954.15	2.75	273.37	988.91	2.63	278.25
SII	351.01	2.82	299.93	381.57	2.85	309.89
SIII	2424.12	39.51	24.08	2232.13	39.57	24.51
SIV	1475.43	31.06	32.21	1369.56	30.97	33.01

**Table 5:** Standard displacement for the 4 samples in the initial state and after the application of accelerated aging.

Standard Force, N	Standard Displacement, mm							
	SI		SII		SIII		SIV	
	Initial	After Accelerated Aging	Initial	After Accelerated Aging	Initial	After Accelerated Aging	Initial	After Accelerated Aging
10	0.315	0.307	0.697	0.791	0.513	0.391	0.714	0.619
20	0.618	0.596	0.903	1.413	0.917	0.619	1.219	0.831
50	1.193	1.181	1.514	1.917	1.693	1.407	2.091	1.512
70	1.671	1.597	1.819	2.419	2.219	1.809	2.714	1.903
100	2.231	2.213	2.279	3.117	2.895	2.501	3.293	2.301
150	3.259	2.915	2.987	4.213	3.817	3.387	4.307	2.583
200	4.175	3.718	3.401	5.301	4.901	4.311	5.491	3.279
